# Online self-management fall prevention intervention for people with multiple sclerosis: a feasibility study protocol of a parallel group randomised trial

**DOI:** 10.1136/bmjopen-2022-061325

**Published:** 2022-07-08

**Authors:** Marie Kierkegaard, Elizabeth Peterson, Susanna Tuvemo Johnson, Kristina Gottberg, Sverker Johansson, Marie Elf, Maria Flink, Charlotte Ytterberg

**Affiliations:** 1 Department of Neurobiology, Care Sciences and Society, Karolinska Institutet, Stockholm, Sweden; 2 Academic Specialist Center, Region Stockholm, Stockholm, Sweden; 3 Department of Occupational Therapy, College of Applied Health Sciences, University of Illinois Chicago, Chicago, IL, USA; 4 Women’s Health and Allied Health Professionals Theme, Karolinska University Hospital, Stockholm, Sweden; 5 School of Education and Learning, Dalarna University, Falun, Sweden

**Keywords:** multiple sclerosis, qualitative research, rehabilitation medicine

## Abstract

**Introduction:**

Falls among people with multiple sclerosis (PwMS) are common and associated with injuries, fear of falling and low health-related quality of life. Considerations of behavioural, environmental, psychological and physical influences (including ambulation status) are needed to meet fall prevention needs for PwMS. Thus, using a codesign process involving key stakeholders a novel online self-management fall prevention intervention was created specifically for ambulatory and non-ambulatory PwMS. The feasibility, acceptability, fidelity and outcome of this complex intervention will be explored. Findings will inform a future full-scale randomised controlled trial.

**Methods and analysis:**

A mixed-method design will be used. Forty-eight PwMS, stratified for ambulation level, will be randomised to control (n=24) or intervention (n=24). Both groups will receive a brochure about fall risk factors and fall prevention. The intervention is group-based (eight PwMS in each group); will be delivered online; and involve six, 2-hour weekly sessions and a booster session 8 weeks after the sixth session. Each intervention group will be led by a trained facilitator. Data collection will be performed at baseline, and after seven and 18 weeks. Outcome measures will capture data on fall prevention behaviours, fear of falling, falls self-efficacy, social and everyday activities, perceived impact of MS and number of falls. Feasibility of recruitment process, data collection procedures, outcome measures, and delivery, and intervention acceptability, fidelity and outcomes will be evaluated. Both quantitative and qualitative methods will be used.

**Ethics and dissemination:**

Ethical approval has been obtained from the Swedish Ethical Review Authority (registration number 2021-04817). Results will be disseminated in peer-review journals, at conferences, research meetings, in social media and through the patient organisation Neuro Sweden.

**Trial registration number:**

NCT04317716.

**Strengths and limitations of this study**

- This study protocol describes the first feasibility evaluation of an online self-management fall prevention intervention for both ambulatory and non-ambulatory people with multiple sclerosis (PwMS).
- A systematic approach is used for evaluation of outcome and mechanism of the intervention.
- The evidence generated from this study will inform refinement of the intervention in advance of a full scale randomised controlled trial.
- The online delivery of the intervention will possibly exclude PwMS with low socioeconomic status and low computer/technical skills.

## Introduction

 Multiple sclerosis (MS) is a chronic inflammatory, demyelinating and neurodegenerative disease with a global prevalence of about 36 per 100 000 population.^1^ The prevalence in women is 2–4 times higher than in men^1^ and the usual onset is between 20 and 40 years of age. The disease is typically progressive in nature with consequences that include an increased risk for falls. Up to 71% of people with MS (PwMS) fall each 6 months.^2^ Those who report a fall in the past year have an 82% probability of falling again in the 6 months after a fall and a 56% probability of sustaining an injurious fall.^2^ Falls among PwMS are associated with injuries, fear of falling and low health-related quality of life.^3^,^5^

Several consequences of MS are known fall risk factors for this population including impaired balance,^6 7^ reduced walking speed^7^ and impaired cognition.^6^ The lack of attention given to behavioural and environmental influences on fall risk for PwMS is a major gap in existing evidence. Another major gap in MS falls research is that few studies have explored influences on fall risk among individuals who are non-ambulatory, that is, only capable of walking a few steps or not at all.^4 8^ Furthermore, most interventions aiming to prevent falls among PwMS have excluded non-ambulatory individuals. To date, only two research teams are addressing the fall prevention needs of non-ambulatory PwMS: ours and a team based in the U.SA.^9^,^11^ The importance of targeting a variety of modifiable risk factors through fall prevention programmes for PwMS has been highlighted.^12^ The need for comprehensive approaches to fall prevention that address physical, environmental and behavioural aspects of falls management has been echoed by Gunn *et al*.^13^ Despite the recognition of the value of attention to diverse influences on fall risk, most fall prevention interventions for PwMS focus only on addressing physical impairments, such as compromised balance.^14^ For PwMS, who live with an unpredictable disease and daily fluctuations in functioning, the benefits of self-management^15 16^ of the multifactorial fall risks have been highlighted.^17 18^ Nevertheless, research on self-management interventions to prevent falls in PwMS is in its infancy. The delivery and settings of previous interventions have been face to face in physical locations^19 20^ supported by online resources^13 21 22^ or web-based without any in real time interactions.^23^ Self-management interventions can be enhanced by digital health technologies such as in real time digital meetings and online learning platforms. For PwMS, such delivery will require less time and no expenses for travelling, reduced impact on fatigue and have the possibility to reach people also in sparsely populated areas.

To address the unique fall prevention needs of ambulatory and non-ambulatory PwMS, we have designed an online self-management fall prevention intervention. The intervention is a complex intervention, given its number of interacting components; the number and difficulty of behaviours required by both those delivering and receiving the interventions; and the number of outcomes addressed.^24^ The intervention was developed informed by findings from our scoping review (manuscript in preparation) and using a codesign method,^25^ based on design thinking,^26 27^ with the goal of enhancing the intervention’s quality and relevance to end users. Codesign can be explained as the process or act of creating with stakeholders, that is, a creative participatory method. Overall, the codesign process (including preplanning, the workshops and the refinement phase) was 9 months in duration; captured feedback from various stakeholders (PwMS, the patient organisation Neuro Sweden and healthcare professionals); and resulted in a multifaceted self-management intervention.

### Aims

The aims of this feasibility study are to examine feasibility, acceptability, fidelity, and outcome of the online, codesigned self-management fall prevention intervention for ambulatory and non-ambulatory PwMS, and to examine feasibility of the recruitment process, the data collection procedures, and the outcome measures. In accordance with the Medical Research Council (MRC) framework,^24^ we will evaluate the study design and the intervention itself.

Specifically, the following research questions (RQ) will be addressed:

1. Is the recruitment process feasible?
2. Are data collection procedures feasible?
3. Is the delivery of the intervention feasible?
4. Is the intervention fidelity consistently maintained during delivery?
5. Are the outcome measures feasible?
6. Is the intervention acceptable for PwMS?
7. Is the intervention acceptable for the facilitators?
8. Are there any differences in outcomes between and within groups?
9. Are there any dropouts and if so, what are the reasons?
10. Are there any adverse events?

## Methods and analysis

### Study design

**Figure 1:**
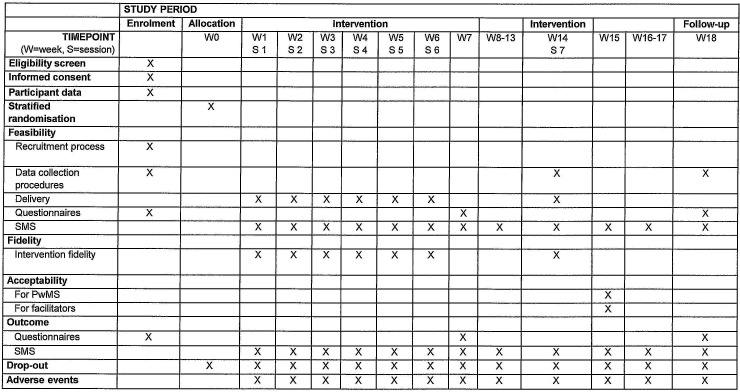
Schedule of enrolment, allocation, intervention and assessments. PwMS, people with multiple sclerosis; SMS, short message service.

This feasibility study will use a mixed-method design consistent with the MRC framework for developing and evaluating complex interventions.^24^ Thus, both quantitative and qualitative methods will be used. See figure 1 for an overview of the study. Findings will inform a future full-scale randomised controlled trial (RCT).

### Patient and public involvement

The intervention is codesigned in close collaboration between PwMS, healthcare professionals, researchers and a facilitator. Furthermore, PwMS representatives from the patient organisation Neuro Sweden are project partners which ensures the patient benefit.

### Participants

Eligible participants are PwMS who fulfil the following inclusion criteria: community dwelling; aged ≥18 years; can independently transfer from bed to wheelchair with or without aids but without assistance of another person; can understand and communicate in Swedish; have ability to use and access to technical devices for online meetings that is, computers or tablets with internet access. The study will recruit 48 PwMS; 24 PwMS will be randomised to participate in the group-based self-management intervention (eight participants in each group) and 24 PwMS will be randomised to the control group.

### Recruitment sites and procedures

Recruitment will take place at the Academic Specialist Centre of Neurology and at the Karolinska University Hospital, Stockholm, Sweden. Outpatients with MS scheduled for an appointment at these sites will be briefly informed about the study as part of their clinical visit and, if interested to participate, asked for permission to provide their contact information to one of the research assistants. Further, advertisements will be published at (1) Academic Specialist Centre of Neurology, Stockholm, Sweden and (2) the patient organisation Neuro Sweden. Those PwMS who are interested to participate will be asked to contact one of the research assistants.

One of the research assistants will screen possible participants for eligibility and provide PwMS who fulfil the inclusion criteria with information about the study. Both oral and written information will be given, and informed consent will be obtained before enrolment in the study. One of the research assistants will perform baseline assessments online and assign participants with an ID number which will be used during baseline and follow-up assessments. After completion of baseline assessments, a person with no further involvement in the study will allocate participants to intervention or control group. Participants (n=48) will be stratified for ambulation level (ambulatory/non-ambulatory) and a 1:1 allocation ratio of blocks of four will be used. Implementation of the random allocation will be done by means of sequentially numbered sealed opaque envelopes.

### Control

Control-group participants will receive a brochure, sent by post, about fall risk factors and fall prevention in addition to the standard MS care and rehabilitation.

### Intervention

The fall-prevention intervention features self-management content designed to enhance the individual’s ability to manage the symptoms, treatment, physical and psychosocial consequences, and/or lifestyle changes intrinsic in living with a long-term condition.^28^ Consistent with the self-management skills described by Lorig and Holman,^15^ the intervention will focus on six core self-management skills, specifically problem solving, decision-making, using resources, partnership with healthcare providers, taking action and self-tailoring. The mechanisms of the intervention are theoretically grounded in Social Cognitive Theory^29 30^ and the pedagogical format of the intervention is theoretically grounded in Universal Design for Learning.^31^ Examples of application of Social Cognitive Theory include observational learning, persuasion and support and opportunities for skill mastery. Universal Design for learning emphasises the importance of designing an intervention to enable participants of various abilities to assimilate the knowledge using different forms of engagement, materials, action and expression. Each of those features are reflected in the intervention.

The self-management fall prevention intervention is group-based and performed online. It comprises six 2-hour weekly sessions and a booster session held 8 weeks after the sixth session, see table 1. The sessions are real-time, face-to-face digital meetings by use of a video platform (Zoom Video Communications). An online learning platform (Canvas, Instructure) is used to share intervention content including assignments to be completed by participants between sessions, for asynchronous activities and communication between participants outside the sessions. The assignments are designed to provide participants with opportunities to practice skills learnt during the intervention. In addition, intervention group participants will also receive the brochure about fall risk factors and fall prevention given to the control group.

**Table 1:** Timeline and content of the self-management fall prevention intervention

Session	The aim is that participants should
Session 1 (week 1)	Be secure in using the online platforms; get to know each other; and build trust.
Session 2 (week 2)	Comprehend the aim of the programme and its structure; share and discuss fall experiences.
Home-assignment	Identify one’s own fall risk situations
Session 3 (week 3)	Understand what an action plan is; initiate ideas about one’s own action plan to prevent falls
Home-assignment	Draft one’s own action plan
Session 4 (week 4)	Finalise action plan
Home-assignment	Test and evaluate strategies in the action plan
Session 5 (week 5)	Follow-up of action plan; if needed revise the action plan; and become aware about adjustment of expectations and demands in daily life to one’s own capacity
Home-assignment	Test and evaluate strategies in the action plan
Session 6 (week 6)	Follow-up of action plan; if needed revise the action plan; and learn about maintenance of motivation for continuous use of action plans
Home-assignment	Continue to use the action plan
Session 7—’booster session’ (week 14)	Follow-up of use of action plan; plan for maintenance of motivation for continuous use of action plans

This feasibility study will include delivery of the intervention in three groups, comprising eight participants. The intervention facilitators, licensed health professionals such as occupational therapists, physiotherapists or nurses, with experience in MS care/rehabilitation and/or fall prevention, will be recruited within the research team and their professional networks. To support programme fidelity, the facilitators will be trained by two researchers (MF, CY) from the research team to deliver the intervention. Each of the three facilitators will deliver one full cycle of the intervention. This will ensure that the outcomes of the intervention are not attributed to an individual facilitator.

### Data collections outcome measures

Online data collections will be performed at baseline, and at follow-ups seven and 18 weeks after the start of the intervention/control period for all participants. Furthermore, participants in the intervention group and the three facilitators will be asked to participate in individual interviews within 1 week after the booster session. In addition, data collections will also be performed during and after each session of the intervention.

Baseline data will include self-reported information on sociodemographic characteristics (eg, age, sex, housing, living situation, work situation, education level); disease-related characteristics (eg, MS severity, time since diagnosis, symptoms, medications and other treatment, other diagnoses, aids, formal/informal care, falls and fall related injuries the previous 3 months); and study expectations. Data on MS severity will be based on the Expanded Disability Status Scale (EDSS)^32^ considering self-reported ambulatory function and categorised as mild (no impairment in walking, ie, ≤EDSS 3.5), moderate (from being able to walk without aid or rest for 500 m to requiring two walking aids to walk about 20 m without resting, ie, EDSS 4.0-6.5), and severe (unable to walk beyond approximately 5m even with aid, ie, ≥EDSS 7).

Outcome measures, collected at baseline and follow-ups seven and 18 weeks after the start of the intervention/control period, will include data on fall prevention behaviours assessed by the Fall Prevention Strategies Survey^33^; a direct measure of fear of falling^34^; falls self-efficacy assessed by the Short Falls Efficacy Scale-International^35^ (ambulatory participants) and the Spinal Cord Injury Fall Concern Scale^36^ (non-ambulatory participants); social and everyday activities assessed by the Frenchay Activities Index^37^; perceived impact of MS assessed by the Multiple Sclerosis Impact Scale^38^ and falls. Falls will be monitored from baseline to the 18-week follow-up via an online short message service (SMS)/fall diary and interview. All study participants will be asked to note their falls, circumstances of the fall, injuries and care seeking. An SMS will be sent once a week (to avoid recall bias) asking ‘Have you fallen within the last week?’ Participants answering ‘yes’ will be contacted by telephone for an interview with questions on circumstances of the fall, injuries and care seeking. Fall is defined as ‘an unexpected event in which the participants come to rest on the ground, floor or lower level’.^39^

### Evaluation plan

#### Assessments of feasibility and intervention fidelity (RQ 1–5)

Data on feasibility of recruitment process (RQ1) will be collected through the research assistants’ registrations of the number of eligible informed participants; number recruited; reason for declining participation and time needed for participant recruitment. Data on feasibility of data-collection procedures (RQ2) will be collected through research assistants’ registrations of number of participants completing each data-collection including registration of falls through SMS/falls diary; number of participants completing each outcome measure and time needed to complete each data-collection.

Data on feasibility of delivery of the intervention (RQ3) and intervention fidelity (RQ4) will be collected through observations of each group session, facilitators’ reflections after each session, and data from the intervention online platform. The observations will be conducted by the researchers in the research team not involved in the delivery of the intervention using a semistructured protocol to assess number of completed sessions, participants’ and facilitators’ activity in sessions, and the extent to which intervention manual was followed. A semistructured interview will explore the facilitators’ reflections of barriers and facilitating factors for following the manual, if applicable reasons for not following the manual, and overall experience of the session. Participants’ online activity between sessions will be collected from automatic registrations of the intervention platform, such as chat activity and uploading of home assignments. The criterium for programme adherence for PwMS will be attendance of at least five of the seven sessions.

Data on feasibility of outcome measures (RQ 5) will be collected through descriptive statistics of floor and ceiling effects, analytic statistics of sensitivity to change, and time taken to complete each data collection.

#### Assessment of acceptability of the intervention (RQ 6–7)

Data on acceptability of the intervention from the perspectives of the PwMS who participated in the intervention (RQ 6) and facilitators (RQ 7) will be collected through interviews conducted by researchers in the research team not involved in the delivery of the intervention. Interviews will be held with each individual participant in the intervention group and each individual facilitator. The semistructured interviews exploring their experience of the intervention will occur within 1 week after the booster session. The interviews with both intervention participants and facilitators will cover views regarding limitations and potential benefits of the intervention, perspectives of intervention mechanisms (eg, creation and use of action plans, group discussions), strengths and weaknesses of the online platform and technology utilised, and overall strengths and limitations of online delivery. Interviews with participants will explore whether the intervention was perceived as acceptable and their overall experience as an intervention participant. Interviews with facilitators will explore facilitators’ experience delivering the intervention and their overall experience as a facilitator.

#### Assessment of differences in outcomes between and within groups (RQ 8)

Data on fall prevention behaviours,^33^ fear of falling^34^ falls self-efficacy,^35 36^ social and everyday activities^37^;and perceived impact of MS^38^ will be collected at baseline and at follow-ups 7 and 18 weeks after the start of the intervention/control period. Falls will be monitored from start of intervention/control period to the 18-week follow-up via SMS/fall diary and interview.

#### Assessments of drop-out rate and reasons, and adverse events (RQ 9–10)

Data on drop-out rate, that is, withdrawals from the study, will be collected (RQ9). Participants who decide to terminate their participation in the feasibility study will be asked to share their reasons for drop-out. Data on potential adverse events of the interventions such as falls and activity curtailment, time-management-related stress experienced when trying to incorporate new skills, learnt from the intervention, into day-to-day life (RQ 10) will be collected by interviews, SMS and falls-diaries.

### Analyses

Qualitative content analysis^40 41^ will be used to analyse data from observations, reflections and semistructured interviews to explore feasibility of intervention delivery, and fidelity and acceptability of the intervention. Descriptive statistics will be used for analyses of feasibility of recruitment process, data collection procedures and outcome measures; and of drop-out rates and reasons for dropout. Descriptive and comparative statistics will be used for analyses of between and within group differences in outcome measures and number of reported falls. There will be no formal hypothesis testing as it is a feasibility study and, thus, not powered for such analyses. We will, however, report change data with estimates and 95% CI. Depending on data level and distribution, parametric or non-parametric analyses will be performed. An a priori statistical analysis plan will be developed, and analyses will be performed and reported in accordance with the Consolidated Standards of Reporting Trials guidelines for pilot and feasibility trials.^42^

### Ethics and dissemination

Information will be given both orally and written, and all participants need to give their signed informed consent before entering the study. Procedures will be conducted in accordance with the Declaration of Helsinki. Ethical approval was obtained for this study from Swedish Ethical Review Authority (registration number 2021-04817). Results of the feasibility study will be disseminated in peer-review journals, at national and international conferences, at research meetings, in social media and through the patient organisation Neuro Sweden. Deidentified data from the feasibility study and additional information, will be made available on reasonable request after publication of results.

## Discussion

The intervention contrasts with the majority of fall preventions for PwMS which heavily address remediation of impairments, such as comprised balance and mobility. Two features of the intervention have great potential to strengthen its ability to meet specific needs of PwMS. First, both ambulatory and non-ambulatory PwMS were deeply involved in the development of the intervention’s content and format. This is important because qualitative studies have demonstrated that PwMS have unique insights regarding their fall prevention needs that can improve fall prevention interventions.^18^ Second, the intervention will use a self-management approach to support day-to-day management of participants’ chronic fall risks. The benefits associated with self-management interventions have been widely documented^16^ and may be particularly important for PwMS who live with an unpredictable disease and daily fluctuations in functioning. The intervention is novel in many ways: it was developed using codesign with PwMS and healthcare professionals; it targets both ambulatory and non-ambulatory PwMS; it focuses on self-management and it is delivered online. The groups participating in this parallel feasibility RCT will, however, not be attention matched which can be seen as a limitation. A strength of our evaluation plan is the adherence to the MRC framework to develop and evaluate the intervention.^24^ The findings will be instrumental to inform refinement of both the study design and the intervention before a future large-scale RCT.
