# Supplementary material for: Online self-management fall prevention intervention for people with multiple sclerosis: a feasibility study protocol of a parallel group randomised trial
Source: BMJ Open. 2022 Jul 8;12(7):e061325. doi: 10.1136/bmjopen-2022-061325 (PMC9272100; doi:10.1136/bmjopen-2022-061325)
Supplement: Supplementary file 1 [file bmjopen-2022-061325.reviewer_comments.pdf]

PEER REVIEW HISTORY

BMJ Open publishes all reviews undertaken for accepted manuscripts. Reviewers are asked to complete a checklist review form (**http://bmjopen.bmj.com/site/about/resources/checklist.pdf**) and are provided with free text boxes to elaborate on their assessment. These free text comments are reproduced below.

ARTICLE DETAILS

**TITLE (PROVISIONAL)**	An online self-management fall prevention intervention for people with multiple sclerosis: a feasibility study protocol of a parallel group randomised trial
**AUTHORS**	Kierkegaard, Marie; Walker Peterson, Elizabeth; Johnson, Susanna Tuvemo; Gottberg, Kristina; Johansson, Sverker; Elf, Marie; Flink, Maria; Ytterberg, Charlotte

VERSION 1 – REVIEW

**REVIEWER**	Bevens, William<br>The University of Sydney, Brain and Mind Centre
**REVIEW RETURNED**	28-Mar-2022

**GENERAL COMMENTS**	I really enjoyed this manuscript and this is very important work. I especially appreciate the mixed-methods approach to feasibility testing and this adds a lot of depth to your work. Glad to see protocols being published.<br><br>Major:<br>Introduction is far too lengthy, and contains material best suited for the methods section. The 2nd and 3rd paragraphs could be combined in my opinion. The final two paragraphs of the introduction contain information I would expect to see in the methods section, not introduction, and this takes away from the narrative of this study. Realistically, page 5 line 15 to line 38 could transferred to the methods section (or omitted) [on re-reading the manuscript: yes, I believe this part of the introduction is much better suited in the Methods section under the 'Intervention' subheading]. What I would like to see is more rationale on is why the authors chose to evaluate a digital health intervention and not a face-to-face intervention. I see the major rationales for this study in two ways:<br>1. The need for self-management interventions to prevent falls in MS that address physical, environmental, and behavioural aspects;<br>2. The delivery of these programs is enhanced through digital health technologies.<br><br>The 2nd point needs to be addressed in the introduction, particularly as there are previous online falls management programs that should be considered (Kannan, 2019), as well as physical activity interventions that investigate falls as secondary outcome (lots of Motl's work from memory).<br><br>Control group: Control groups can be very difficult to design for self-management programs so I have a few questions:<br>1. What is the purpose of a control group for this feasibility study? If you are using this feasibility study as a way to determine sample size for the future effectiveness/efficacy study by measuring

	outcome differences between intervention and control, a sample size justification should be presented. This is dependent on your primary outcome measure, which is not listed but seems equal weighting is put on all research questions.<br><br>2. Why is the control group a usual-care group and not wait-list? It is likely that undertaking a self-management program alone will impact a participant in important ways (self-efficacy, mental health QoL etc) and this makes comparisons with a usual care group that did not participate in a self-management program difficult. Particularly with outcome measures such as social and everyday activities;and perceived impact of MS, I would suspect merely taking an online course would impact these measures. Worth considering.<br><br>3. Is there any checking for whether participants in the usual care control group undertake a self-management program during the 18 week study period? If so, worth excluding in my opinion.<br><br>Minor:<br>Abstract - Line 10: Considerations of...<br><br>Methods: in eligibility, what is the rationale for " can independently transfer from bed to wheelchair with or without aids but without assistance of another person" and is this a commonly used criteria within falls research?<br><br>It would be great to see actual outcomes from the co-design process i.e., what was decided or changed due to this process. This process is rarely described in the literature and I believe it would be very valuable to be able to see actual outcomes from this process.<br><br>What is the rationale behind the 18-week data point for data collection?<br><br><br>Concluding remarks:<br>I really enjoyed this protocol and I will be excited to read the results. It is very important work and this is a well-considered study. I would suggest making changes to the introduction as described here to further establish the rationale.

**REVIEWER**	Gunn, H<br>Plymouth University
**REVIEW RETURNED**	08-Apr-2022

**GENERAL COMMENTS**	Many thanks for submitting this interesting and well laid out protocol. The intervention is relevant and will potentially add to the evidence base/ be helpful for pwMS should it be successful. I have a couple of comments for you to consider<br><br>Dropouts: Can you clarify what your criteria will be for programme adherence? What happens if people miss a session?<br><br>Intervention: I would welcome some clarification on the nature of the online platform- what will it look like, what will it contain- Whilst your intervention appears to be predominantly ‘real-time/ face to face, the later narrative suggests an element of asynchronous content/ activity.<br><br>Data analyses: More data on the comparative analyses would be

	helpful. Will you develop an a priori statistical analysis plan? Reference to the CONSORT guidelines for pilot and feasibility trials could be useful here. Have you set any criteria to determine progression to a full effectiveness trial?<br><br>Strengths and limitations: I would suggest an acknowledgement that the groups are not attention matched would be reasonable- given the nature of the intervention and outcomes, this could be important (especially considering the behavioural aspects of the programme).

VERSION 1 – AUTHOR RESPONSE

Reviewer: 1
Dear Dr Bevens,
Thank you for your kind words and your suggestions and comments on our manuscript. We have addressed them as described below.

Major:
Introduction is far too lengthy and contains material best suited for the methods section. The 2nd and 3rd paragraphs could be combined in my opinion.

- *Thank you for your suggestion, we have now combined the 2nd and 3rd paragraphs.*

The final two paragraphs of the introduction contain information I would expect to see in the methods section, not introduction, and this takes away from the narrative of this study. Realistically, page 5 line 15 to line 38 could transferred to the methods section (or omitted) [on re-reading the manuscript: yes, I believe this part of the introduction is much better suited in the Methods section under the 'Intervention' subheading].

- *Thank you for your suggestion, we have revised the manuscript accordingly and have now omitted a major part of the description on how the intervention was developed.*

What I would like to see is more rationale on is why the authors chose to evaluate a digital health intervention and not a face-to-face intervention. I see the major rationales for this study in two ways: 1. The need for self-management interventions to prevent falls in MS that address physical, environmental, and behavioural aspects; 2. The delivery of these programs is enhanced through digital health technologies. The 2nd point needs to be addressed in the introduction, particularly as there are previous online falls management programs that should be considered (Kannan, 2019), as well as physical activity interventions that investigate falls as secondary outcome (lots of Motl's work from memory).

- *Thank you for these comments. We agree with the reviewer and have therefore added the following sentences in the introduction (the new second paragraph):*
  "The delivery and settings of previous interventions have been face-to-face in physical locations{Finlayson, 2009 #18;Hugos, 2016 #90} supported by online resources{Gunn, 2018 #86;Gunn, 2019 #72;Gunn, 2021 #85} or web-based without any in real time interactions{Kannan, 2019 #89}. Self-management interventions can be enhanced by digital health technologies such as in real time digital meetings and online learning platforms. For PwMS, such delivery will require less time and no expenses for

travelling, reduced impact on fatigue, and have the possibility to reach people also in sparsely populated areas."

Control group: Control groups can be very difficult to design for self-management programs so I have a few questions:

1. What is the purpose of a control group for this feasibility study? If you are using this feasibility study as a way to determine sample size for the future effectiveness/efficacy study by measuring outcome differences between intervention and control, a sample size justification should be presented. This is dependent on your primary outcome measure, which is not listed but seems equal weighting is put on all research questions.

2. Why is the control group a usual-care group and not wait-list? It is likely that undertaking a self-management program alone will impact a participant in important ways (self-efficacy, mental health QoL etc) and this makes comparisons with a usual care group that did not participate in a self-management program difficult. Particularly with outcome measures such as social and everyday activities;and perceived impact of MS, I would suspect merely taking an online course would impact these measures. Worth considering.

- *Thank you for your questions. We have included a control-group as we want to test the feasibility of all steps that will be taken in a potential future full-scale RCT, e.g., time taken to recruit participants and for outcome assessments, the randomization procedure, the text message delivery, and follow-up etc. We therefore argue that there is no need for a wait-list control group. We are not conducting this feasibility study to determine a future sample size per se, therefore no primary outcome measure. Our intention is rather to pilot our outcome measures and see how they change over time (validity) both between and within groups. We argue that this will give valuable information and together with previous research on reliability and responsiveness to change will inform future sample size calculations. Thus, even though we include assessments of differences between- and within-groups in our feasibility RCT, our focus is feasibility, acceptability, and fidelity of this complex intervention and not effectiveness.*

- *We do, however, acknowledge the imbalance in attention given to the intervention and control group and have therefore added the following sentence in the discussion section: "The groups participating in this parallel feasibility RCT will, however, not be attention matched which can be seen as a limitation."*

3. Is there any checking for whether participants in the usual care control group undertake a self-management program during the 18 week study period? If so, worth excluding in my opinion.

- *Thank you for this relevant question. We will, however, not collect any information about this during the feasibility study, but will consider this if/when we move to a full-scale RCT.*

Minor:
Abstract - Line 10: Considerations of...

- *Thank you, we have now changed this in the abstract.*

Methods: in eligibility, what is the rationale for " can independently transfer from bed to wheelchair with or without aids but without assistance of another person" and is this a commonly used criteria within falls research?

- *Thank you for question. The rationale for* " can independently transfer from bed to wheelchair with or without aids but without assistance of another person" is that our self-management intervention is intended for ambulatory and non-ambulatory PwMS. *We drew from the the Expanded Disability Status Scale (EDSS), an internationally accepted method of quantifying disability/disease severity in MS to develop a criteria to operationalize the definition of "non-ambulatory" for the purposes of our study. EDSS scores range from 0 (no*rmal function) to 10 (death due to MS). *At EDSS stage 7,5 the PwMS can independently transfer from bed to wheelchair with or without aids but without assistance of another person. Above that stage PwMS rely completely on other persons when it comes to transfers and locomotion. We believe that our self-management intervention will likely be most relevant for PwMS who have some mobility. Many people who live with MS and who use wheelchairs and/or scooters full time walk short distances, for example during transfers, and we have therefore chosen this eligibility criteria. This criteria is not commonly used within falls research because fall prevention research involving PwMS who are "non-ambulatory" is in its infancy*

It would be great to see actual outcomes from the co-design process i.e., what was decided or changed due to this process. This process is rarely described in the literature, and I believe it would be very valuable to be able to see actual outcomes from this process.

- *Thank you very much, and yes, we do plan to describe this process and are currently working on such a manuscript.*

What is the rationale behind the 18-week data point for data collection?

- *We chose to include a three-month follow-up (i.e., after the sixth session) as we wanted to have a longer follow-up than directly after the last (sixth) session of the intervention and did not want to have it directly after the booster-session.*

Concluding remarks:
I really enjoyed this protocol and I will be excited to read the results. It is very important work and this is a well-considered study. I would suggest making changes to the introduction as described here to further establish the rationale.

- *Thank you for these kind words and we hope you are satisfied with our revised manuscript.*

Reviewer: 2

Dear Dr Gunn,

Thank you for your kind words and your comments on our manuscript. We have addressed them as described below.

Dropouts: Can you clarify what your criteria will be for programme adherence? What happens if people miss a session?

- *Thank you for this relevant question. First, we would like to clarify that with dropouts we mean PwMS who withdraw from the study. This has been inserted in the first sentence in the paragraph under the subheading "Assessments of dropout rate and reasons, and adverse events (RQ 9-10)" which now reads: "Data on dropout rate, i.e., withdrawals from the study, will be collected (RQ9)."*
  The criterium for programme adherence will be attendance of at least five of the seven sessions. We have added this sentence last in the second paragraph under the subheading "Assessments of feasibility and intervention fidelity (RQ 1-5)".

Intervention: I would welcome some clarification on the nature of the online platform- what will it look like, what will it contain- Whilst your intervention appears to be predominantly 'real-time/ face to face, the later narrative suggests an element of asynchronous content/ activity.

- *Thank you for this comment. You are right that we use both real-time face-to-face digital meetings for the sessions (Zoom) and a learning platform (Canvas) for asynchronous content/ activity. The learning platform is used for the assignments between sessions, for sharing materials and gives the participants the opportunity to interact and chance to communicate/chat with each other outside the session.*
- *We have clarified this by adding information in the second paragraph under the subheading "Intervention" as described below:*
  "The sessions are real-time, face-to-face digital meetings by use of a video platform (Zoom Video Communications, Inc.). An online learning platform (Canvas, Instructure Inc.) is used to share intervention content including assignments to be completed by participants between sessions, for asynchronous activities and communication between participants outside the sessions".

Data analyses: More data on the comparative analyses would be helpful. Will you develop an a priori statistical analysis plan? Reference to the CONSORT guidelines for pilot and feasibility trials could be useful here. Have you set any criteria to determine progression to a full effectiveness trial?

- *Thank you for these valuable comments. Yes, we will develop an a priori statistical analysis plan and we will adhere to the CONSORT guidelines for pilot and feasibility trials. As we will perform a feasibility study, formal hypothesis testing is not relevant. We will, however, report change data with estimates and 95% CI. Depending on data level and distribution, parametric or non-parametric analyses will be performed. We have added* the following *sentences in the paragraph under the subheading "Analyses" which we hope will clarify:*
  "Descriptive and comparative statistics will be used for analyses of between and within group differences in outcome measures and number of reported falls. There will be no formal hypothesis testing as it is a feasibility study and, thus, not powered for such analyses. We will, however, report change data with estimates and 95% confidence intervals (CI). Depending on data level and distribution, parametric or non-parametric analyses will be performed. An a priori statistical analysis plan will be developed, and analyses will be performed and reported in accordance with the CONSORT guidelines for pilot and feasibility trials {Eldridge, 2016 #92}."
- *As we have not finalised the criteria to determine progression to a full effectiveness trial, we have chosen not to include any information on this in the study protocol. We are currently discussing these issues and we believe that our qualitative interviews with*

participants in the intervention group will likely be very informative and important. Thus, we are at this point of time reluctant to provide criteria stating that X% of this or that need to be fulfilled for progression to a full-scale RCT.

Strengths and limitations: I would suggest an acknowledgement that the groups are not attention matched would be reasonable- given the nature of the intervention and outcomes, this could be important (especially considering the behavioural aspects of the programme).

- *Thank you, we agree and have therefore added the following sentence in the discussion section: "The groups participating in this parallel feasibility RCT will, however, not be attention matched which can be seen as a limitation."*

VERSION 2 – REVIEW

**REVIEWER**	Bevens, William<br>The University of Sydney, Brain and Mind Centre
**REVIEW RETURNED**	31-May-2022

**GENERAL COMMENTS**	I appreciate the consideration the authors have put into the original and revised manuscript. Very excited to read the results of the feasibility study once it has been published. Good luck!
